# Draft Genome Sequence of a Diazotrophic, Plant Growth–Promoting Rhizobacterium of the *Pseudomonas syringae* Complex

**DOI:** 10.1128/genomeA.01023-16

**Published:** 2016-09-22

**Authors:** Cheryl L. Patten, Haeyoung Jeong, Andrew J. C. Blakney, Natalie Wallace

**Affiliations:** a Department of Biology, University of New Brunswick, Fredericton, New Brunswick, Canada; b Super-Bacteria Research Center, Korea Research Institute of Bioscience and Biotechnology, Daejeon, Republic of Korea

## Abstract

We report here the draft genome sequence of *Pseudomonas syringae* GR12-2, a nitrogen-fixing, plant growth–promoting bacterium, isolated from the rhizosphere of an Arctic grass. The 6.6-Mbp genome contains 5,676 protein-coding genes, including a nitrogen-fixation island similar to that in *P. stutzeri*.

## GENOME ANNOUNCEMENT

A well-studied plant growth–promoting pseudomonad, isolated from the roots of an Arctic grass by its ability to fix atmospheric dinitrogen, was originally identified as *Pseudomonas putida*, based on phenotypic characteristics (1, 2). However, phylogenetic analyses of the 16S rRNA and four housekeeping genes showed that it forms a monophyletic group with the *P. syringae* complex of plant pathogens and shares 99% nucleotide sequence identity with the 16S rRNA genes from the *P. syringae* pathovars (3). We have sequenced the genome of *P. syringae* GR12-2 to further characterize the evolutionary relationships among the *P. syringae* strains and to identify genes that may function in plant growth promotion.

Genomic DNA was extracted from *P. syringae* GR12-2 using the Wizard genomic DNA purification kit (Promega, Madison, WI, USA). A genomic DNA shotgun library with an insert size of approximately 460 bp was prepared and 101-cycle paired-end sequencing was performed (National Instrumentation Center for Environmental Management, Seoul, Republic of Korea) using an Illumina HiSeq 2000 platform (CASAVA version 1.8.2). A total of 113,670,590 read pairs were generated, totaling 11,480,729,590 bases. The sequence reads were assembled *de novo* using CLC Genomics Workbench version 4.9 (CLC bio, Cambridge, MA, USA). Scaffolds were generated *in silico* using SSPACE version 1.1 (4), and gaps were automatically filled by running four iterations of IMAGE (5). Automatic open reading frame (ORF) prediction and functional annotation were carried out by the Rapid Annotation using Subsystems Technology server (6, 7) and the automatic Prokaryote Genome Annotation Pipeline (NCBI, Bethesda, MD, USA).

The *P. syringae* GR12-2 genome sequence consists of 74 contigs (maximum contig length, 557,998 bp; *N*_50_, 181,449 bp) totaling 6,601,350 bp with a GC content of 58.3% and contains 11 rRNA and 55 tRNA genes. Of the 5,676 predicted protein-coding sequences, 713 with annotated functions do not have orthologs in the genomes of *P. syringae* pathovars DC3000, B728a, and 1448A. These encode proteins for nutrient uptake and metabolism, environmental interactions, and gene regulation. A 49-kb cluster of 59 genes encoding proteins required for nitrogen fixation is similar in sequence (average 72% amino acid sequence identity) and synteny to a nitrogen-fixing island in *P. stutzeri* A1501 (8). A similar nitrogen-fixation island is present in *P. azotifigens* and other members of the *P. stutzeri* group (9), but not in the available genome sequences of any other pseudomonad. In *P. syringae* GR12-2, the nitrogen-fixation cluster is found in a different region of the genome from that in *P. stutzeri*, suggesting acquisition via horizontal gene transfer. While the capacity to fix nitrogen does not contribute directly to plant growth promotion (2), an understanding of the genes that are unique to a plant-beneficial strain of the *P. syringae* group will provide insight into the evolution of a mutualistic lifestyle.

### Accession number(s).

This whole-genome shotgun project has been deposited at DDBJ/EMBL/GenBank under the accession number LGSI00000000. The version reported in this paper is the first version, LGSI01000000.
